# Influence of Insert Brand and Culture Method on Ciliary Activity and Epithelial Cell Types in Human Nasal Air–Liquid Interface Cell Cultures

**DOI:** 10.3390/life15060958

**Published:** 2025-06-14

**Authors:** Patricia Celkova, Emilie Seydoux, Susan De Groof, Loretta Müller

**Affiliations:** 1 Division of Pediatric Respiratory Medicine and Allergology, Department of Pediatrics, Inselspital, Bern University Hospital, 3010 Bern, Switzerland; celkova22@uniba.sk (P.C.); emilie.seydoux@insel.ch (E.S.); susan.degroof@insel.ch (S.D.G.); 2 Lung Precision Medicine (LPM), Department for BioMedical Research (DBMR), University of Bern, 3008 Bern, Switzerland; 3 Department of Pediatrics and Adolescent Medicine, Jessenius Faculty of Medicine in Martin, Comenius University in Bratislava, University Hospital Martin, 036 59 Martin, Slovakia; 4 Multidisciplinary Center for Infectious Diseases, University of Bern, 3012 Bern, Switzerland; 5 Graduate School for Cellular and Biomedical Sciences, University of Bern, 3012 Bern, Switzerland

**Keywords:** air–liquid interface, cell culture, nasal epithelial cells, cell culture insert, respiratory epithelium

## Abstract

Cultures of primary human nasal epithelial cells (hNECs) differentiated at the air–liquid interface (ALI) represent a sophisticated and widely used model of the human upper respiratory epithelium. Despite the availability of various cell culture insert types and the well-established understanding that different culture media influence the cell culture characteristics, the possible impact of the insert brand remains rather underexplored. We cultured hNECs from nineteen healthy adult donors on three distinct brands of commercially available inserts—Corning^®^ Transwell^®^, CELLTREAT^®^, and ThinCert^®^—and compared the ciliary activity and cellular composition of the cultures using high-speed video microscopy and flow cytometry, respectively. Additionally, we employed an alternative method of hNEC culture setup—the inverted condition—wherein the hNECs were seeded on the basal side of the insert with the idea to avoid mucus accumulation. Our results show that ciliary activity and cell type composition did not differ between insert types for both culture conditions. However, we found a higher ciliary beat frequency and a lower active (ciliated) area in the inverted setup compared to the conventional setup across all three insert brands. These findings indicate that all three mentioned insert types yield comparable cell cultures.

## 1. Introduction

The human respiratory system relies on defensive and self-cleaning mechanisms, particularly the mucociliary transport system [1]. The respiratory epithelium varies from pseudostratified in large airways to columnar and cuboidal in small airways [2], comprising, among others, ciliated, mucus-producing, and basal cells [3,4]. The mucociliary transport system, essential for airway clearance, requires the coordinated action of airway surface fluid production and beating of motile cilia [5]. This system’s effectiveness is crucial for the entrapment and expulsion of particulate matter and pathogens from the airways [6].

Respiratory epithelial cells and their dysfunction are central to the pathogenesis of various lung disorders such as bronchial asthma, COPD, and bronchogenic carcinoma [2], as well as rare inherited disorders like primary ciliary dyskinesia (PCD) [7] and cystic fibrosis (CF) with impaired ciliary motility or affected mucus viscosity, respectively. In addition, they are also suspected to play a role in idiopathic lung fibrosis development [8]. Given the limitations of animal models, including ethical concerns and costs, in vitro models are increasingly crucial for studying respiratory diseases and testing treatments [9,10]. In vitro models, particularly cell cultures grown at the air–liquid interface (ALI), are valuable for their ability to simulate physiological conditions of the human airway, promoting a mucociliary phenotype [11,12]. The unique characteristic of ALI cell culture involves seeding proliferating respiratory epithelial cells on an artificial semi-permeable membrane (called an insert or transwell) submerged in a cell culture medium. Once confluent, the cells are exposed to air on the apical side and still supplemented with nutrients and growth factors on the basolateral side. This resembles the in vivo situation in the lungs with nutrient supplementation from the blood on the basolateral side and exposure to the air on the apical side.

ALI cell cultures are suitable for studying respiratory epithelium physiology, modeling respiratory diseases [9], studying infections [9,13], mimicking inhalative delivery for drug testing [14], and assessing the toxicity of inhaled substances [15,16,17]. They are also crucial for the diagnostics of primary ciliary dyskinesia by significantly reducing the need for repeated brushings in patients [18], eliminating potential secondary factors affecting the analysis of cilia function (e.g., cilia loss due to viral infection), and they provide more material, which ends in a clearer result [18].

Despite the widespread use of ALI cell cultures in respiratory research, differences in protocols and material suppliers introduce potential sources of variability. Although the impact of different culture media on resulting culture compositions is already recognized [19,20,21], the influence of insert types remains relatively underexplored. Recently, Brocke et al. [22] compared two commercially available cell culture inserts from CELLTREAT^®^ and Corning^®^ Transwell^®^, finding no statistically significant differences in the cell differentiation outcomes of the resulting hNECs grown at the ALI.

In this study, we conducted a comparative analysis of primary hNEC cultures from nineteen healthy non-smoking adult donors, cultivated at the ALI using three distinct commercially available culture inserts: Corning^®^ Transwell^®^, CELLTREAT^®^, and ThinCert^®^. Our investigation aimed to assess their impact on the resulting ciliary activity and cellular composition. Additionally, we explored an alternative method of hNEC culture setup—the inverted condition—wherein hNECs were seeded on the basal side of the insert, supplemented with media from the apical side and exposed to air from the basolateral side with the idea to avoid mucus accumulation.

## 2. Materials and Methods

All procedures were performed in compliance with relevant laws and institutional guidelines and have been approved by the ethics committee of the Canton Bern (Kantonale Ethikkomission Bern), Switzerland (project identification code 2018-02155, 01/2019). We obtained written informed consents from all participants.

### 2.1. Human Nasal Epithelial Cell (hNEC) Cultures

Nasal brushing samples were collected from nineteen healthy non-smoking adult donors after obtaining informed consent and ensuring privacy. Samples were processed as previously published [18] with slight modifications. Briefly, after obtaining hNECs by nasal brushings from the inferior nasal turbinate area of both nostrils, the cells were proliferated over two passages in expansion media (PneumaCult-Expansion-Plus complete medium, Stemcell Technologies, Vancouver, BC, Canada, #05040) and 200’000 cells were seeded onto the apical sides of three uncoated insert types (Corning^®^ Transwell^®^ polyester membrane inserts, #3460, Sigma-Aldrich, Merck & Cie, Buchs, Switzerland; CELLTREAT^®^ Permeable Cell Culture Inserts #230621; ThinCert^®^ Cell Culture Inserts, #665641, greiner bio-one; for details of the composition, see Table 1) placed in 12-well cell culture plates (Falcon 12-well plate, #353043).

For the inverted setup, inserts were placed upside down in a 6-well plate (Falcon 6-well plate, #353046) and the same number of cells as for the conventional setup were added in 250 μL of media to the basal side of the membrane. With the lid closed, but ensuring the lid did not touch the cell suspension, the plates were placed in the incubator at 37 °C and 5% CO_2_. After 4 h, excess medium with unattached cells was aspirated, surfaces were washed using PBS with MgCl_2_ and CaCl_2_ (PBS^++^; Sigma-Aldrich, D8662-500 mL), and inserts were transferred into a 12-well plate with 500 μL of expansion media in the apical chamber and 1 mL in the basolateral chamber.

**Figure 1:**
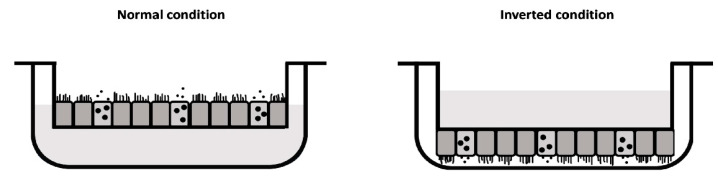
Schematic comparison of the different culture conditions: normal/conventional versus inverted. In the inverted condition, hNECs are seeded on the basal side of the semi-permeable membrane and are supplemented with ALI media in the upper compartment while exposed to air from the lower compartment. Black dots should depict mucus.

Subsequently, all culture conditions were maintained in submerged conditions, with expansion media changed three times per week. One day after reaching full confluence, cultures were exposed to the ALI by exposing the side of the cells to air and changing to ALI medium (PneumaCult ALI complete medium, Stemcell Technologies, cat#05001) in the chamber without cells (700 μL basolateral for conventional and 500 μL apical for inverted conditions). The medium was changed three times per week. Once mucus production began (approximately after 1 week at the ALI), cultures were washed once a week with 500 µL PBS^++^. After at least 28 days at the ALI, fully differentiated hNEC cultures were used for experiments. For the schematic comparison of the conventional and inverted ALI conditions, see Figure 1.

### 2.2. High-Speed Video Microscopy (HSVM) for Analysis of Ciliary Activity

For the analysis of ciliary activity, top-view recordings of intact differentiated hNEC culture inserts were taken using an inverted Olympus IX73 (Tokyo, Japan) light microscope equipped with a high-speed C-MOS camera (FLIR 3.2 MP Mono Grasshopper3 USB 3.0 Camera, Sony IMX252 chip) and a cellVivo incubation system preheated to 37 °C following a previously established protocol [23]. For each insert, videos of the same five spots (top, right, bottom, left, center) were taken (10× phase contrast objective, 2 s, 300 frames/second, resolution of 480 × 640 pixels). The analysis was conducted using Cilialyzer software (version v1.3.0) [24]. Briefly, recordings were preprocessed (binning, image stabilization, mean subtraction), the threshold was set to 0.5, and mean ciliary beating frequency (CBF) and active area (AA), representing the total area where ciliary motion was detected, were calculated. CBF and AA values were calculated and averaged for individual inserts and then insert types in both culture conditions.

### 2.3. Flow Cytometry for Analysis of Cell Culture Composition

**Figure 2:**
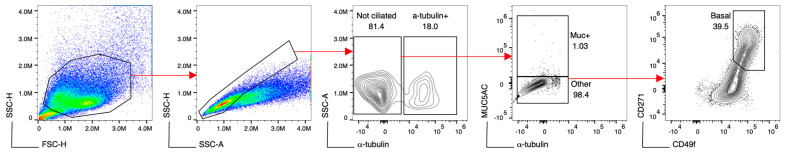
Representative gating strategy for flow cytometry analysis of hNECs. Cells were gated for singlets. Ciliated cells were identified as α-Tubulin+, mucus-producing cells as α-Tubulin- and MUC5AC+, and basal cells as α-Tubulin- and CD271+ CD49f+. Percentages indicate the frequency of each gated population in a representative donor sample. Colors indicate intensity (one blue dot corresponds to one cells, green-yellow-red indicate increase in cell number), black boxes represent gated cells, red arrows show the chosen cells for the next graph, Data were acquired on a Cytek Aurora flow cytometer and analyzed using FlowJo v10.8.

hNECs from four donors undergoing all culture conditions were detached from the inserts by adding 1 mL trypsin on the apical side for 10 min at 37 °C. Using this technique, cells detached without the need to scrape them off, improving their viability. Cells were subsequently washed with PneumaCult ALI complete medium and resuspended in 500 µL FACS buffer (PBS without MgCl_2_ and CaCl_2_ (D8537-500 mL, Sigma-Aldrich) with 1% BSA (A9647-100G, Sigma-Aldrich) and 1mM EDTA (AM9260G, Invitrogen, Thermo Fisher Scientific, Zurich, Switzerland)). Cells were transferred into a round-bottom 96-well plate and centrifuged at 300 g for 5 min at 4 °C. The supernatant was discarded by flicking the plate, and cells were surface-stained for viability (eBioscience Fixable Viability Dye eFluor 450, Thermo Fisher, #65-0863-18; dilution 1:1600) and for MUC5AC-Alexa Fluor 488 (Novus Biologicals, Lucerna-Chem AG, Zug, Switzerland, #NBP2-54448AF488; dilution 1:400), acetylated α-Tubulin-Alexa Fluor 546 (Santa Cruz Biotechnology, Labforce, Muttenz, Switzerland, #sc-23950; dilution 1:200), CD271 (NGFR)-APC (BioLegend, Amsterdam, The Netherlands, #345107; dilution 1:800), and CD49f (ITGA6)-APC-Cy7 (BioLegend, #313627; dilution 1:400). Unstained controls were used as negative controls. After incubation for 20 min on ice, cells were washed once with FACS buffer and fixed in 100 µL Fixation buffer (ThermoFisher, #00-8222-49) for 15 min on ice. Cells were washed once with FACS buffer and resuspended in 200 µL FACS buffer until acquisition. Data was acquired on the Cytek Aurora Spectrum Analyser and analyzed using FlowJo version 10.8. Cells were gated as singlets, followed by identification as α-Tubulin+ (ciliated cells), MUC5AC+ (mucus-producing cells), or CD271+ CD49f+ (basal cells). The gating strategy is shown in Figure 2.

### 2.4. Statistical Analysis

We analyzed cell cultures from seventeen donors for ciliary beat frequency (CBF) and active area (AA), and from four donors for flow cytometry-based cell composition analysis. Two donors who participated in the initial CBF/AA experiments were rebrushed for the subsequent flow cytometry analysis. CBF and AA data were normally distributed, while the number of replicates for the flow cytometry data was too low for a normal distribution (tested with the Kolmogorov–Smirnov or D’Agostino–Pearson test). One insert (Corning^®^ Transwell^®^ in the inverted condition) from a single donor was excluded from statistical analysis of ciliary activity due to differentiation failure, and one insert (COSTAR^®^ in the inverted condition) from a single donor was excluded from FACS statistical analysis due to a low count of cells in the analyzed sample. Therefore, for the analysis of CBF and AA, repeated measures one-way ANOVA with Geisser–Greenhouse correction followed by Tukey’s multiple comparisons test was used to compare the insert types within each culture condition, and a paired t-test was used to compare the culture conditions within each insert type. For cell type distribution, the Kruskal–Wallis test was used for the comparison, due to the low number of repetitions and missing values. A value of *p* ≤ 0.05 was defined as statistically significant. Statistical analysis was performed using GraphPad Prism v.10.0.0.

## 3. Results

### 3.1. Study Population

We used hNEC cultures from 17 donors for the CBF and AA analysis, and from 4 donors for the analysis of the cell culture composition (details in Table 2). The sex distribution was quite equal, and the age range covered the whole span of adulthood, excluding the elderly age range (>60 years old).

### 3.2. No Morphological Differences Between Insert Types and Culture Conditions

**Figure 3:**
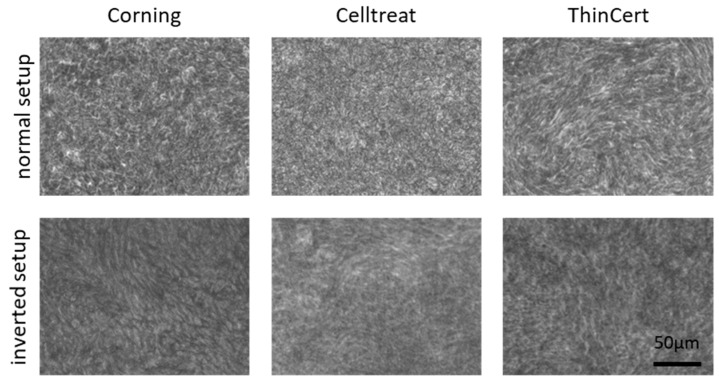
Top-view images of the cell cultures grown on the different insert types and with the different setups, showing no morphological differences. These representative images from one donor were retrieved from the video frame sequence.

The qualitative analysis of the transmission images retrieved from the video sequences and the observations carried out during cultivation and experiments showed no differences between the three insert types (Figure 3). All conditions showed a similar homogeneous morphology.

### 3.3. Comparison of Physical Characteristics of Insert Types

The membrane material for both Corning^®^ Transwell^®^ and ThinCert^®^ is polyethylene terephthalate (PET), whereas for CELLTREAT^®^, it is polyethylene (PE). Pore diameter, culture area, and membrane diameter are comparable across all inserts, with only minor differences. Pore density varies quite considerably among the compared inserts, with Corning^®^ Transwell^®^ having the highest and ThinCert^®^ the lowest, while CELLTREAT^®^ does not provide this information. The data are summarized in Table 1.

### 3.4. Ciliary Activity Differs Between Culture Conditions, but Not Between Insert Brands

**Figure 4:**
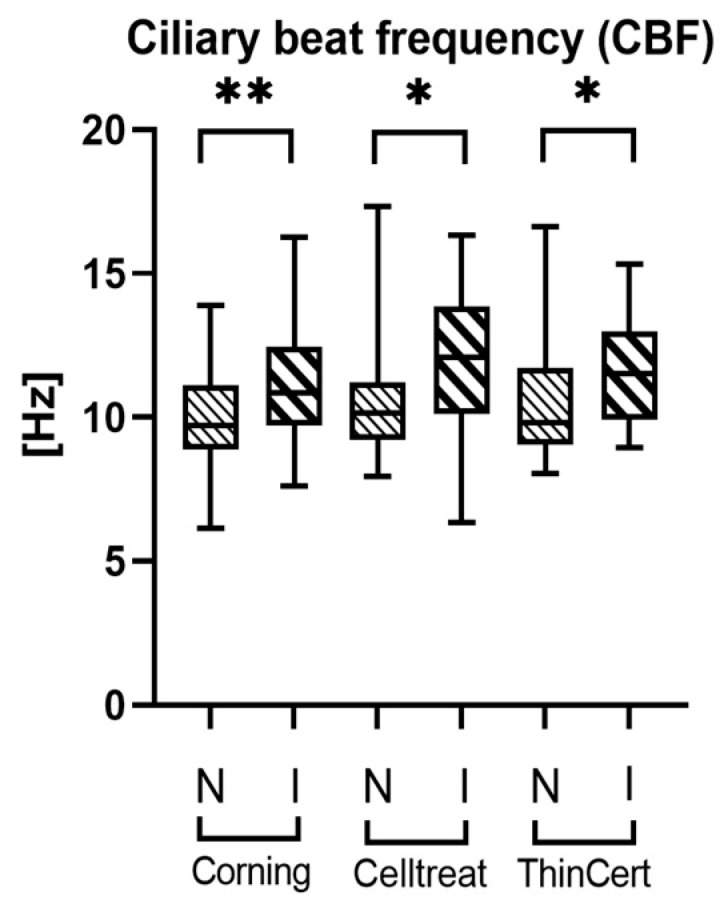
Comparison of mean ciliary beat frequency (CBF) values across all tested insert brands in normal and inverted culture conditions. Significantly higher mean values of CBF are observed in inverted (I) culture conditions compared to the normal (N) setup. Data are shown in a box and whisker plot with min to max range, *n* = 17. * *p* < 0.05 and ** *p* < 0.01 using a paired t-test to compare culture conditions within each insert type. There are no statistically significant differences between the insert types within each culture condition.

**Figure 5:**
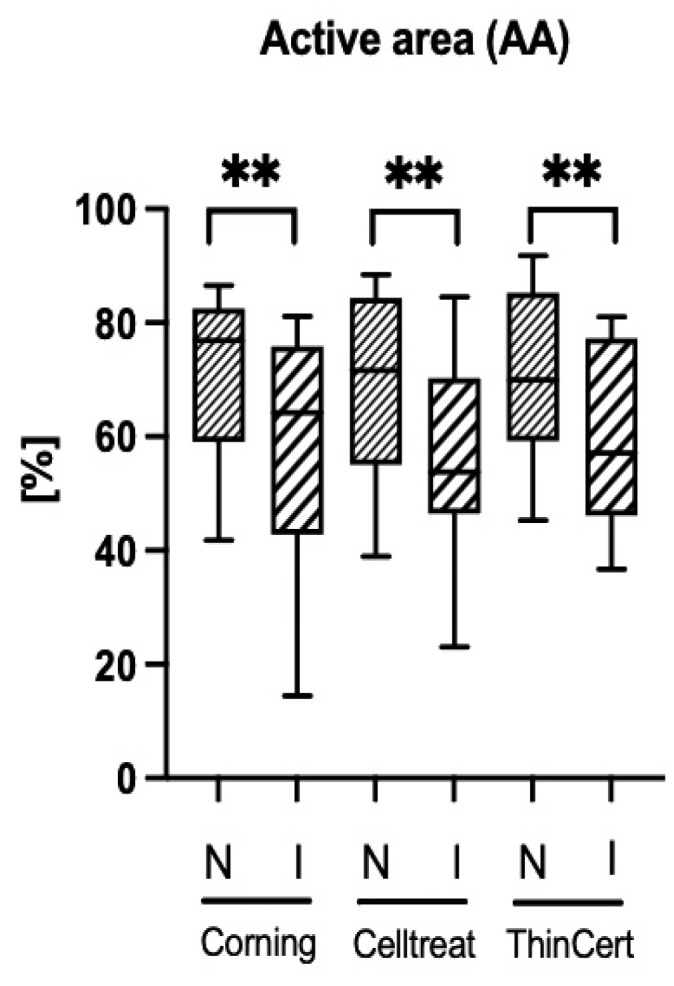
Comparison of mean active area (AA) ratios across all tested insert brands in normal and inverted culture conditions. A significantly lower ratio of AA is observed in inverted (I) conditions compared to the normal (N) setup. Data are shown in a box and whisker plot with min to max range, *n* = 17. ** *p* < 0.01 using a paired t-test to compare culture conditions within each insert type. There are no statistically significant differences between the insert types within each culture condition.

We observed no significant difference in CBF (Figure 4) or AA (Figure 5) between the tested insert brands. However, for all three insert brands, we found the CBF to be significantly higher (Figure 4) and the AA lower (Figure 5) in the inverted culture conditions compared to normal culture conditions. We observed that both culture conditions showed identically circular mucus transport.

### 3.5. No Differences in Cell Culture Composition

**Figure 6:**
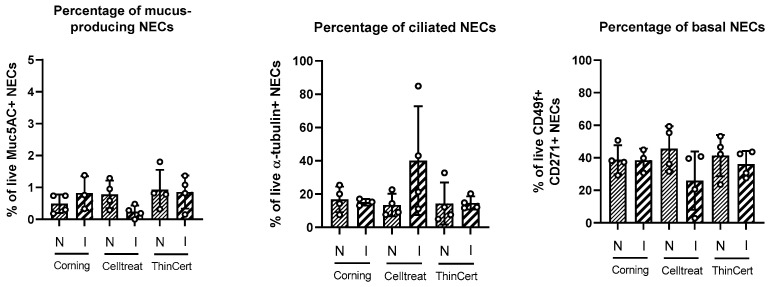
Comparison of cellular composition in different cell culture conditions. No differences are found between insert brands or culture conditions. Data are shown in a scatter dot plot with the line at mean, standard deviation (vertical lines), and all data points shown (as circles).

Cell cultures from a subset of donors (*n* = 4) were analyzed for their differentiation into different epithelial cell types, namely mucus-producing cells (stained for MUC5AC), ciliated cells (stained for acetylated α-tubulin), and basal cells (stained for CD49f (NGFR) and CD271 (ITGA6)). We observed no significant differences between insert brands or culture setups (Figure 6). The approximate percentage for each cell type was 40% for basal cells, 20% for ciliated cells, and 1% for mucus-producing cells. In this analysis, hNEC ALI cultures grown on CELLTREAT^®^ in inverted conditions exhibited the most variable ciliated cell count compared to the other insert brands and culture setup, in which the cell counts were more consistent.

## 4. Discussion

Our study showed no differences in the CBF, AA, and cell composition of ALI hNEC cultures when comparing Corning^®^ Transwell^®^, CELLTREAT^®^, and ThinCert^®^ cell culture inserts. However, the inverted culture conditions resulted in a higher CBF and a lower AA for all tested insert brands.

Our results are consistent with Brocke et al. [22], who found a difference neither in CBF nor in AA between Corning^®^ Transwell^®^ and CELLTREAT^®^. In our study, we also included ThinCert^®^, which did not differ from the other two brands. While Brocke et al. included only 3 donors and recorded CBF at room temperature, resulting in CBF values between 2 and 5 Hz, we included 17 donors, recorded at 37 °C, and obtained CBF values between 5 and 17 Hz. Additionally, AA is not temperature-dependent, but most probably highly software-dependent: Brocke et al. and our group used similar culture protocols and insert types, but we obtained very different values for AA (0–30% in their study versus 17–85% in our study) [22]. A higher percentage of AA is indicative for more ciliated cells and thus we see this as a marker for optimally differentiated cell cultures. Differences in temperature for recording and the used software do unfortunately not allow for comparing absolute values between different studies. However, as each study used a similar analysis approach and both found no effect of the insert type on CBF and AA, we can conclude that all three brands can be used for cell culture comparably without significantly affecting ciliary activity parameters.

As already mentioned above, we included these brands of inserts due to their interindividual comparability based on material and dimensional properties. As per dimensional properties, we focused on membrane diameter, culture area, pore diameter, and pore distribution. Pore density varies the most between the tested insert brands and could be a hypothetical source of variability in ciliary activity and cell type composition in resulting cultures. Redman et al. mentioned that ThinCerts have a lower pore density, which may slow the nutrient flow and thus result in differences in epithelial morphology [19]. However, despite these differences, we did not observe significant variations in CBF, AA, or cell type composition across the three insert brands. This suggests that within the tested range, pore density alone may not be a critical factor in determining the functional and structural outcomes of ALI hNEC cultures. Yet, another parameter to potentially consider is pore distribution, which could be crucial for cell culture insert selection, especially in inserts with larger pore diameters. However, this information is not commonly provided by manufacturers. From our center’s observation, the pore distributions of all three insert types appear to be similarly random.

Despite no difference being found between insert brands, we observed significant differences in CBF and AA when comparing inverted and normal culture conditions, where the average CBF was higher and the AA lower in the inverted cultures. We hypothesized that this culture composition would simplify mucus elimination from the culture inserts due to the absence of a spatial limitation on the insert’s lateral walls. Similar strategies have been explored in airway epithelial models, where inverted cultures led to a thinner, more evenly distributed mucus layer, potentially reducing ciliary load and mucus viscosity and enhancing CBF. Moreover, inverted ALI cultures maintained epithelial integrity and differentiation, suggesting that this method could provide a more physiologically relevant model of mucociliary function [25]. However, based on our visual inspection of the cell cultures, we cannot confirm this. In future studies, the production of mucus, the thickness of the mucus layer, and the barrier integrity of the cell layer should be investigated.

We speculate that potential changes in the microenvironment (e.g., humidity or CO_2_ level) between the two culture conditions may result in a lower ratio of ciliated cells in the inverted culture conditions. Furthermore, we hypothesize that the higher CBF in the inverted culture condition is a compensatory effect of the cultures for the lower AA to keep the mucociliary transport constant. However, this hypothesis needs further investigations as there is not much published about these aspects. One study mentions higher CBF in the smaller airways of mouse lung slices, which are known to have less ciliated cells compared to the larger airways [26], findings that can be interpreted to support our hypothesis.

The cellular composition did not significantly differ between insert types or between different culture conditions. This confirms our findings of similar AAs between the insert types, which would correspond to similar proportions of ciliated cells between the insert types, but contrasts with the significant difference in AA observed between the culture conditions. A potential source of this inconsistency might be the lower number of included donors for flow cytometry analysis of the cellular composition. Moreover, hNECs—and especially ciliated cells—were found to be very sensitive to trypsinization, yielding a higher percentage of non-viable cells positive for α-tubulin, which may partly explain the low proportion of ciliated cells observed, although additional factors such as donor variability or handling loss cannot be excluded. These findings may also reflect the higher sensitivity of functional assays like active area measurements in detecting ciliary coverage, compared to surface-marker-based flow cytometry, especially in studies with a low sample size and potential cell loss during detachment. Beyond its impact on ciliary activity, the inverted culture setup may offer practical advantages for high-throughput applications. The reduced media volume required in the apical compartment, along with the downward-facing orientation of the epithelial layer, could help minimize contamination risk. These attributes may make the inverted method particularly well suited for larger-scale studies where consistency, efficiency, and accessibility are critical. However, the initial seeding process in the inverted configuration can be more technically demanding and should be taken into consideration. Furthermore, the inverted culture method may also contribute to overcoming some of the technical challenges commonly associated with ALI cultures. In standard conditions, mucus accumulation, limited accessibility to the cell layer, and sensitivity to environmental factors can make long-term maintenance difficult. By reorienting the culture and potentially reducing mucus buildup, the inverted setup may improve the robustness of epithelial differentiation. This could enhance the overall reproducibility and feasibility of ALI models, making them more accessible for broader use in in vitro respiratory research. However, depending on the aim of the study, e.g., for apical nebulization of drugs, the conventional setup of observation with the cilia pointing up may still be the setup of choice.

Based on the statistically relevant number of donors for the analysis, we conclude that inverted conditions have a significant influence on the CBF and AA in the resulting cultures. Conversely, we state that the three investigated insert brands result in cell cultures with similar CBFs, AAs, and cellular compositions, and can be used interchangeably for studies involving those endpoints.

## 5. Conclusions

As there was no significant difference found in ciliary activity parameters or epithelial cell type distribution between the used insert brands, these findings allow us to conclude that all three insert brands can be used for cell culture comparably without significantly impacting the resulting cultures.

Inverted culture conditions compared to conventional setups resulted in a significantly higher CBF and lower AA, suggesting a lower ciliated cell count, although this was not confirmed by the cellular composition analysis in our experiments. However, based on the high statistical relevance of the observed differences in ciliary activity, we may conclude that these conditions lead to cultures with different characteristics.

## Figures and Tables

**Table 1:** Overview of physical characteristics of the different insert types.

	Corning^®^ Transwell^®^	CELLTREAT^®^	ThinCert^®^
Material	Polyethylene terephthalate	Polyethylene	Polyethylene terephthalate
Pore diameter	0.4 µm	0.4 µm	0.4 µm
Culture area	1.12 cm^2^	1.11 cm^2^ *	1.131 cm^2^
Pore density	4 × 10^6^/cm^2^	not provided	2 × 10^6^/cm^2^
Membrane diameter	12 mm	11.91 mm *	12.07 mm

* Taken from [18] since not provided by manufacturer.

**Table 2:** Characteristics of the study population.

	Sex (Male/Female)	Mean Age (Years)	Age Range (Years)
**CBF/AA analysis**	10/7	34.8	24–60
**Cell culture composition**	2/2	33.8	25–46

## Data Availability

The original contributions presented in this study are included in the article. Further inquiries can be directed to the corresponding author.
